# Land cover matters to human well-being

**DOI:** 10.1038/s41598-021-95351-6

**Published:** 2021-08-05

**Authors:** Chao Li, Shunsuke Managi

**Affiliations:** Urban Institute and School of Engineering, Kyushu University, 744 Motooka, Nishi-ku, Fukuoka, 819-0395 Japan

**Keywords:** Environmental impact, Social evolution, Risk factors, Environmental economics, Environmental impact

## Abstract

People migrate from rural to urban areas. In the meantime, the benefits of staying in greener areas are also known. People’s preferences might be different by area that is composed of several land types. If so, the effect of particular land cover on human well-being is different spatially. The spatial analysis is required to formulate effective land-use policies. Here we show that urban land, water, and grassland are positively related to human well-being, whereas bare land is negatively associated in Japan. A 1 ${\mathrm{m}}^{2}$ increase in the area of urban land per capita in a city is equivalent to an about 346 USD increase in the individual annual income of all the people in the city. Additionally, monetary values of areas of water, crops, and bare land per capita are 102, − 30, and − 268 $\mathrm{USD}/\mathrm{Capita }{\mathrm{m}}^{2}$. Furthermore, the spatial context matters to the relationship between land cover and human well-being. This paper investigates the monetary values of several land types and their spatial variability, which provides insights to make better usage for land cover.

## Introduction

People migrate from rural to urban areas^1,2^. In the meantime, the benefits of staying in greener areas are also known^3,4^. The areas where people locate are related to their preference of particular land type^5,6^. People’s preferences might be different by area that is composed of several land types. If so, the effect of particular land cover on human well-being is different spatially. The spatial analysis is required to formulate effective land-use policies. Almost 91.7% Japanese population live in cities in 2019, and the proportion will increase to roughly 94.7% by 2050^1,2^. Japan still has an abundant natural environment, whose forest rate is 67.26%, even with so high urbanization rate. Nevertheless, an increase in the urbanization rate does not mean that many more people would live in cities, although the population in cities is still expanding. For instance, the Japanese population is 126.9 million in 2019, while it will become 116.7 million in 2050^2^. Land cover in Japan is changing^7,8^, due to the decrease of the population from 2010^2^ and urbanization^1^, similar to counter-urbanization^9^. However, the impacts of the land cover change, especially the urban land cover, on human well-being in Japan are unclear in the terminal stage of urbanization. Besides, the impacts of the land cover changes on human well-being are spatially different because of the scarcity value and the spatial contexts. To sum up, correct understanding of the impacts of land cover change in the process of urbanization is an essential issue to improve human well-being^10,11^, to formulate effective land-use policies^12,13^.

The indicators of human well-being are various in previous studies because the relationships between land cover and human well-being have been long investigated^10^. Some studies take subjective well-being (SWB) as the direct indicator of human well-being^14^, including life satisfaction^15–17^, happiness^18,19^ and Cantril’s ladder^5,10,20^. Other studies utilize mental health^3,21^, self-reported health^22^, morbidity^23^, poverty^24^, among others, as the indirect indicators. Among these indicators, life satisfaction is generally taken as the indicator of human well-being^10^. Accordingly, we choose life satisfaction as the SWB indicator in this study. Currently, the single dimension life satisfaction question is widely applied to evaluate overall human well-being. In this study, life satisfaction is a numerical score from 1 (completely dissatisfied) to 5 (completely satisfied), in response to the question: overall, how satisfied are you with your life?

The relationship between green space and SWB is the main direction to be analyzed because green space, or natural environment, is considered an essential factor to provide ecosystem service^25–27^. Natural land cover is positively associated with human well-being^18,28,29^, because it provides more ecosystem service^30^, such as reducing air pollution^17,31^, relief of mental stress^32^, among others. The monetary value of green space is estimated according to the relationship between green space and SWB^5,18,33^. Compared with green space, only several studies take other land types into account^6,24,33^. Abandoned land in cities is negatively related to SWB due to its adverse impacts on mental and physical health^33^. Previous studies reveal that abandoned land, such as vacant land, can cause anxiety, stigma, among others^34^. In the study regarding European Union, the urban land, road, and mine are also positively related to the SWB, similar to natural land cover^6^, because such land types are the environments of daily human life. The green space in different land-use types is discussed in the Great Tokyo Area because of scarcity value^5^. Additionally, land cover changes, including built-up area, water and aquaculture, also have effects on poverty due to flood risk, which is an indirect indicator of human well-being^24^. These previous studies indicate that the marginal willingness to pay (MWTP) should be higher when the natural land cover is scarcer^5,6^. Therefore, the urban land would be desired when it is insufficient compared with the population, although this expectation has been long ignored. In this study, we detect the relationships between SWB and ten types of land cover, including urban land, using life satisfaction as the indicator of SWB.

Furthermore, the relationship between land cover and human well-being with spatial contexts is not deeply analyzed. In order to illustrate spatial variability, difference-in-differences and grouped regressions by administrative regions are utilized in previous studies^6,29,35^. The difference-in-differences method uses location dummy variables of each region^4,35^, while grouped regression method divides the total sample into several subsamples and regresses them individually^6^. Nevertheless, these two methods assume the subsamples are spatially independent, so they are not as much as spatial regression models to reduce spatial heterogeneity. To further solve the spatial heterogeneity, spatial models such as spatial autoregressive model (SAR), spatial error model (SEM), spatial lag of X model (SLX), and geographically weighted regression (GWR) are widely performed on other topics^36^. Especially, GWR is typically a local model to solve the spatial heterogeneity^37^, which is analog to group regression, dividing the total sample into several subsamples but by the spatial matrix on the basis of the distances among regions^38^. The relationship between land cover and SWB by the spatial context in Japan is illustrated by performing GWR in this article.

In addition, the previous studies mainly focus on the individual-level data to avoid ecological fallacy or to provide information for choosing living environments^4,33,35^. For individuals, SWB is higher when there is more green space in people’s living environment. Some previous studies take buffers as the living environment with a certain distance, such as 1 km, 1.5 km among others^5,33^, while others use the administrative regions as people’s living environment^3,21,32^. In fact, the relationship between SWB and land cover, especially urban land, remains unclear at the city level, which is meaningful to policymakers. In this study, the relationship between SWB and land cover is spatially illustrated.

To estimate the monetary values of each land type, the life satisfaction approach (LSA) or SWB method is widely applied in previous studies^39–41^. Income affects SWB, shown in empirical economic analysis^42,43^. Land cover change in the living environment is associated with SWB^10,21^. To offset the effects of land cover changes, income would also alter, assuming no variation in human well-being. The alteration of income is considered the monetary value of land cover change^44^. In the light of the LSA and spatial analyses, the spatial distributions of the monetary values of land cover are demonstrated in this study.

## Materials and methods

### Materials

#### Human well-being data in Japan

Sub-prefecture-level average subjective well-being (SASWB) is applied as the dependent variable. There are 47 prefectures in Japan, and every prefecture has several cities or wards, called sub-prefecture regions, in this article. At first, we obtain the individual SWB data, which indicates human well-being^10,14^, in the surveys from 2015 to 2017. Over 300,000 people are sampled from all over Japan, and we receive roughly 247,105, 130,821, and 100,803 valid answer sheets in 2015, 2016, and 2017 respectively. Then, we use the postcodes to track the respondents’ addresses and ultimately to find which sub-prefecture regions the respondents belong to. Finally, we calculate the means of SWB and some other control variables of each sub-prefecture region.

**Figure 1:**
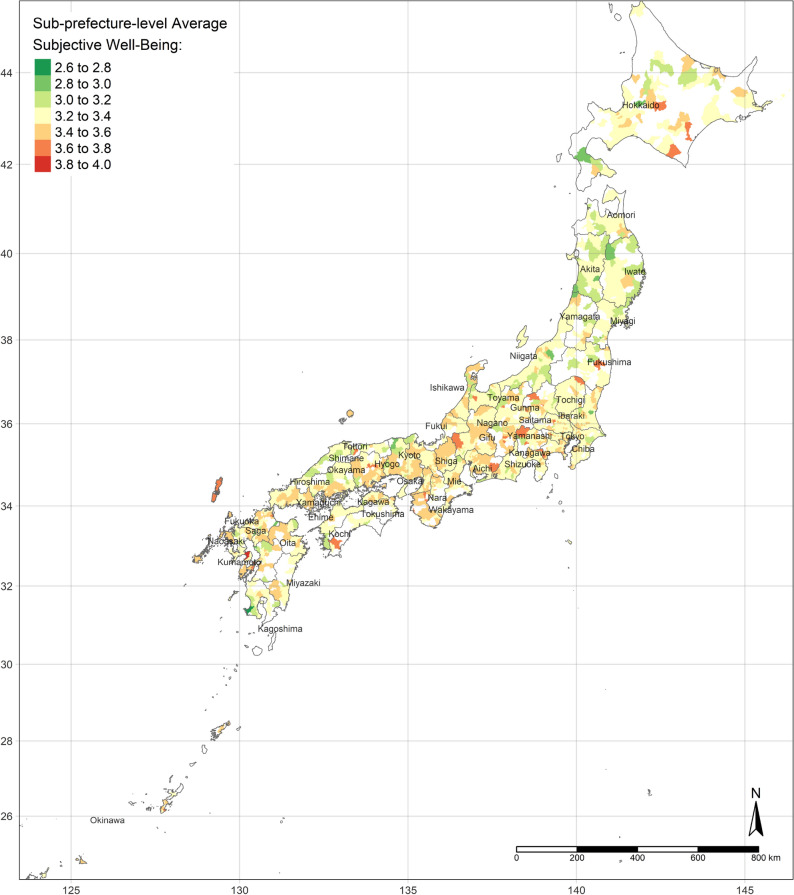
Sub-prefecture-level average SWB. (Figure is created by R 4.0.4, https://cran.r-project.org/bin/windows/base/old/4.0.4/).

In 1830 sub-prefecture regions, we receive fully completed questionnaires. However, we cannot put the cities and wards into regressions without at least 30 completed questionnaires, according to the smallest sample size of t-distribution. Therefore, 1234 sub-prefecture regions’ data are exploited in the analyses (Table S1: Data statistic summary, Fig. 1: SASWB Spatial Distribution).

We claim that the ethics review committee for Kyushu University, Japan approved all experimental protocols for the survey, and all methods are carried out according to the relevant guidelines and regulations. All survey methods were carried out following relevant guidelines and regulations. At the beginning of the survey, respondents are informed about the survey’s aim and their rights to voluntarily participate. All respondents provided informed consent before response the questionnaire.

#### Land cover data

We employ the nationwide land cover dataset with 30 m resolution from the Japan Aerospace Exploration Agency (JAXA), which is generated through remote sensing data of Landsat-8 from 2014 to 2016 (land cover dataset in detail: https://www.eorc.jaxa.jp/ALOS/en/lulc/lulc_index.htm). This study focuses on the spatial heterogeneity of the relationship between human well-being and land cover. Given that the panel land cover dataset is unavailable during survey periods, the spatial models could also demonstrate the spatial variability of the relationship. The land cover dataset includes ten land cover categories, which are water, urban area, rice paddy, crops, grassland, deciduous broadleaf forest (DBF), deciduous needle forest (DNF), evergreen broadleaf forest (EBF), evergreen needle forest (ENF), and bare land.

Areas of 10 land types per capita of sub-prefecture regions are taken as the land cover variables in this study. Using the boundaries of each sub-prefecture region from the Japanese government, the sub-prefecture-level total areas of each land type are calculated. Areas of each land type per capita are the total areas divided by the population of the sub-prefecture regions in 2015. The area per capita depicts both the composition and variety of land types in these cities, compared with total areas and percentages of land types^3,21^.

#### Other control variable

Twenty-four other sub-prefecture-level variables are controlled in the analyses. In the survey, the frequency of feeling high levels of stress and the frequency of feeling low levels of stress are asked, and the respondents should select one choice from 5 (always) and 5 (never). Additionally, the ease of stress relief, living environment comfort, living environment safety, community attachment, and self-reported health are investigated. Moreover, the respondents should also select one choice from 5 (the best feeling) to 1 (the worst feeling). Job, education background, and individual annual income are acquired in the survey. Moreover, the percentage of males, the percentage of the population from 45 to 64 years old, the percentage of the population over 65 years old, and the population density are controlled, provided by Japan government (Table S2: Data sources and detailed information of variables).

### Methods

To deeply dig out the role of spatial heterogeneity on the relationship between SASWB and land cover, five regression models, including four global models and one local model, are applied in our study. They are ordinary least squares (OLS), SAR, SEM, SLX^45^, and GWR, respectively^38,46^. The global models are OLS, SAR, SEM, and SLX, and the local model is GWR^36^. This study uses the simplest model, the OLS, at first. However, the OLS model strongly assumes that every observation is spatially independent, but the other four models accept the spatial dependency of the variables. The SAR assumes the spatial dependency among the dependent variable. The SEM assumes that the error terms or regions are spatially correlated. The SLX assumes a specific region’s SASWB is associated with its neighbors’ independent variables. All of these assumptions might exist but be ignored by the OLS. Additionally, the residuals of the OLS might be spatially clustered rather than randomly distributed, which can be examined by Moran’s I test. To solve this problem, the GWR is performed. The GWR divides the total sample into several subsamples using a specific bandwidth and regresses them^38^, respectively, based on Tobler’s first law of geography. Because subsamples are not the same, the coefficients in each regression may be different. In this way, the GWR is globally non-stationary but locally stationary^38^, illustrating spatial variability of the relationship between land cover and SASWB.

#### OLS model

The OLS model is the most basic regression model among all models mentioned in this article^46^. It explores the relationships between independent variables and a dependent variable directly, and the general form is as follows:

1 $${SASWB}_{i}= {\beta }_{0}+ {{\varvec{\beta}}}_{1}{{\varvec{X}}{^{\prime}}}_{i}+ {\varepsilon }_{i}$$

where ${SASWB}_{i}$ represents average SWB of sub-prefecture region $i$, ${{\varvec{X}}}_{i}$ represents a vector of independent variables of sub-prefecture region $i$, ${\varepsilon }_{i}$ represents the error term, and ${\beta }_{0}$ and ${{\varvec{\beta}}}_{1}$ are parameters to be estimated. Unlike previous studies, SASWB is taken as the dependent variable, which is a continuous variable. Although individual SWB is a discrete variable, not strictly fitting into OLS, SASWB is reasonable in this research.

OLS model has two strong assumptions that the observations and error terms are independent^47^. However, there is no sub-prefecture region absolutely isolated in Japan, owing to the well-developed transportation system. In other words, the observations have spatial correlations, ignored by the OLS model. Thus, we also utilize the other four models, SAR, SEM, SLX, and GWR, to examine the spatial relationships.

#### SAR model

The SAR model assumes that one observation’s dependent variable is associated with its neighbors’ dependent variable. In our study, a sub-prefecture region’s average SWB is related to the average SWB of the regions surrounding it^48^. SAR is shown as follows:

2 $${SASWB}_{i}= {\beta }_{0}+ \rho {{\varvec{W}}}_{i}{{\varvec{N}}{\varvec{E}}{\varvec{S}}{\varvec{W}}{\varvec{B}}\boldsymbol{^{\prime}}}_{i} + {{\varvec{\beta}}}_{1}{{\varvec{X}}{^{\prime}}}_{i}+ {\varepsilon }_{i}$$

where ${{\varvec{W}}}_{i}$ represents a vector of spatial weights of neighbor regions of sub-prefecture region $i$, ${{\varvec{N}}{\varvec{E}}{\varvec{S}}{\varvec{W}}{\varvec{B}}}_{i}$ represents a vector of SASWB of neighbor sub-prefecture region $i$, and $\rho$ is the spatial lag parameter to be estimated. To obtain each observation’s spatial weight vectors, we use the queen method^49^. In the queen method, two polygons are considered as contiguity if they share one point. In addition, the data should be ignored in the SAR model if the observation does not have any neighbors due to missing data or islands themselves.

#### SEM model

The SEM model hypothesizes that there is spatial dependence in the variables ignored by the OLS model^50^. Thus, the error term in OLS is decomposed into two parts, the spatially related error term and the spatially unrelated error term. In our study SEM model is:

3 $${SASWB}_{i}= {\beta }_{0} + {{\varvec{\beta}}}_{1}{{\varvec{X}}{^{\prime}}}_{i}+ \lambda {{\varvec{W}}}_{i}{{\varvec{\epsilon}}\boldsymbol{^{\prime}}}_{i}+ {\eta }_{i}$$

where ${{\varvec{\epsilon}}}_{i}$ represents the part of error term with a spatial correlation of sub-prefecture region $i$, ${\eta }_{i}$ represents the other part of error without spatial correlation of sub-prefecture region $i$, and $\lambda$ is the spatial correlation parameter to be estimated.

#### SLX model

The SLX assumes that the one observation’s dependent variable is related to its neighbors’ independent variables^51^. In fact, the SLX model is another OLS model that takes neighbors’ weighted independent variables into account. The SLX is denoted by:

4 $${SASWB}_{i}= {\beta }_{0} + {{\varvec{\beta}}}_{1}{{\varvec{X}}{^{\prime}}}_{i}+{\varvec{\theta}}{{\varvec{W}}}_{i}{{\varvec{N}}{\varvec{E}}{\varvec{X}}\boldsymbol{^{\prime}}}_{i}+ {\varepsilon }_{i}$$

where ${{\varvec{N}}{\varvec{E}}{\varvec{X}}}_{i}$ represents a vector of independent variables of neighbor regions of sub-prefecture region $i$, and ${\varvec{\theta}}$ is a vector of spatial lag parameters to be estimated.

#### GWR model

The GWR is a typical local model because it does not assume that the relationships between the dependent and independent variables are spatially stationary^38,52,53^. Simply speaking, the GWR model divides the total sample into several subsamples by the spatial contexts of observations. Then, every parameter is estimated in each subsample based on general regression models. Therefore, the parameters spatially vary because they are derived for each location, respectively. However, in the GWR model, the essential issue is the smallest sample size of the subsamples, decided by the bandwidth widely used in R project. The root mean square prediction error for the GWR model is the key indicator to decide the bandwidth. The GWR model is shown as follows^38^:

5 $${SASWB}_{i}= {\beta }_{i0} + \sum_{j=1}^{m}{{\varvec{\beta}}}_{ij}{{\varvec{X}}\boldsymbol{^{\prime}}}_{ij}+ {\epsilon }_{i}, i=1, \dots , n$$

where ${SASWB}_{i}$ represents average SWB of sub-prefecture region $i$, ${\beta }_{i0}$ represents the intercept of sub-prefecture region $i$, ${{\varvec{\beta}}}_{ij}$ represents a vector of parameters in the regression with the $j$ th subsample of sub-prefecture region $i$, $m$ represents the number of subsamples, $n$ represents the number of observations in each subsample, and ${\epsilon }_{i}$ represents the error term. The bandwidth selection and parameters estimation can be achieved using package “GWmodel” in R project. The coefficients of GWR are estimated as follows:

6 $${{\varvec{\beta}}}_{ij} = {[{{\varvec{X}}}^{T}{{\varvec{W}}}_{ij}{\varvec{X}}]}^{-1}{{\varvec{X}}}^{T}{{\varvec{W}}}_{ij}{\varvec{Y}}$$

where ${{\varvec{\beta}}}_{ij}$ represents a vector of parameters in the regression with the $j$ th subsample of sub-prefecture region $i$, ${{\varvec{W}}}_{ij}$ represents the spatially weighted matrix to estimate the coefficients with the $j$ th subsample of sub-prefecture region $i$.

#### Marginal willing to pay (MWTP) of land cover

MWTPs of land types represent these environmental goods’ monetary values, which are difficult to assess. The SWB method is operated, as many previous studies^10,41^. The impacts of environmental change on SWB are offset by the effects on SWB of the individual income variation, assuming nothing else alters except these two variables^39^. MWTP could be explained as the variation in income associated with changes in each unit area of a particular land type per capita in our study. The MWTP is calculated as follows:

7 $${MWTP}_{k}= \frac{{Imp\cdot LC}_{k}}{Imp\cdot Inc} \times Inc$$

where ${MWTP}_{k}$ represents the monetary value of land type $k$, $Imp\cdot Inc$ represents the impacts of individual income on SWB, ${Imp\cdot LC}_{k}$ represents the impacts of the area per capita of land type $k$ on SWB, $Inc$ represents the average individual annual income. Notably, we have to use the terminology “impacts” rather than “coefficients” because, in SAR and SEM models, the coefficients of independent variables are not partial derivation^45^. There is a function “impacts” in R project to calculate both direct and indirect impacts of the independent variables on the dependent variable in SAR and SEM models. In SLX and GWR model, the coefficients can be used because these two models are based on the OLS model.

## Results

Table 1 illustrates a part of the estimated parameters in the OLS, SAR, SEM, and SLX models (Table S3: Full regression results). Average individual annual income is positively associated with SASWB in all four models. Namely, living in the sub-prefecture regions with the higher average individual income, people have higher SWB. Besides, the relationships between SASWB and the area of water per capita as well as the area of urban land per capita are positive. In contrast, the association of SASWB with the area of bare land per capita is opposite in all four models. It indicates that people prefer the cities with more urban land and water but with less bare land. The relationship between SASWB and the area of crops per capita is negative only in the SEM and SLX models. Intriguingly, as shown by the SLX model, even though the area of grassland per capita of the sub-prefecture regions is not significantly related to their SASWB, the relationship between the area of grassland per capita of their neighbor regions and SASWB is significantly positive. The SAR model should be rejected directly, based on Lagrange Multiplier diagnostics for spatial dependence in the OLS model. Compared with Eqs. (1) and (2), if the spatial lag parameter $\rho$ is not significant, there is no difference. In these four models, according to AIC, the SLX model is the best model, and its adjusted R^2^ is 49.2%, higher than the adjusted R^2^ of the OLS model (47.6%). GWR as the local model shows a dominant advantage in AIC (− 2242), but adjusted R^2^ (49.0%) is still lower than the SLX model. According to the statistical test and interpretation of each model, the SLX and the GWR are taken as the main models in further analyses.

**Table 1:** Partial regressions results.

	Dependent variable: SASWB				
	OLS model	SAR model	SEM model	SLX model	
				X	Lag X
Average individual annual income (1 million JPY)	0.017*** (0.006)	0.017*** (0.006)	0.017*** (0.006)	0.015** (0.006)	0.003 (0.011)
Area of water per capita (ha/capita)	0.411*** (0.141)	0.411*** (0.139)	0.425*** (0.141)	0.374** (0.147)	− 0.069 (0.187)
Area of urban land per capita (ha/capita)	0.849** (0.393)	0.846** (0.387)	0.879** (0.403)	1.266** (0.550)	− 0.443 (0.742)
Area of crops per capita (ha/capita)	− 0.078 (0.050)	− 0.078 (0.050)	− 0.085* (0.051)	− 0.109* (0.063)	0.119 (0.087)
Area of rice paddy per capita (ha/capita)	0.060 (0.082)	0.062 (0.081)	0.063 (0.083)	0.155 (0.102)	− 0.115 (0.151)
Area of grassland per capita (ha/capita)	0.013 (0.020)	0.013 (0.019)	0.004 (0.020)	− 0.001 (0.021)	0.047** (0.021)
Area of DBF per capita (ha/capita)	0.005 (0.013)	0.005 (0.013)	0.006 (0.013)	0.005 (0.015)	− 0.005 (0.022)
Area of DNF per capita (ha/capita)	0.016 (0.022)	0.016 (0.022)	0.015 (0.023)	0.003 (0.026)	0.037 (0.040)
Area of EBF per capita (ha/capita)	0.045 (0.039)	0.043 (0.039)	0.044 (0.040)	0.050 (0.055)	− 0.007 (0.076)
Area of ENF per capita (ha/capita)	− 0.004 (0.017)	− 0.004 (0.017)	− 0.005 (0.018)	− 0.008 (0.021)	− 0.002 (0.030)
Area of bare land per capita (ha/capita)	− 1.086*** (0.375)	− 1.086*** (0.370)	− 1.020*** (0.381)	− 0.980** (0.464)	− 0.992 (0.680)
Spatial lag parameter $\rho$		− 0.002			
Spatial correlation parameter $\lambda$			0.088**		
Observations	1,234	1,234	1,234	1,234	
Akaike information criterion (AIC)	− 2225.843	− 2,223.985	− 2,228.206	− 2229.969	

X in the SLX model represents the variables of sub-prefecture regions themselves, while Lag X represents the variables of their neighbors.

(Full regressions results are shown in Table S3).

*p < 0.1, **p < 0.05, ***p < 0.01.

**Figure 2:**
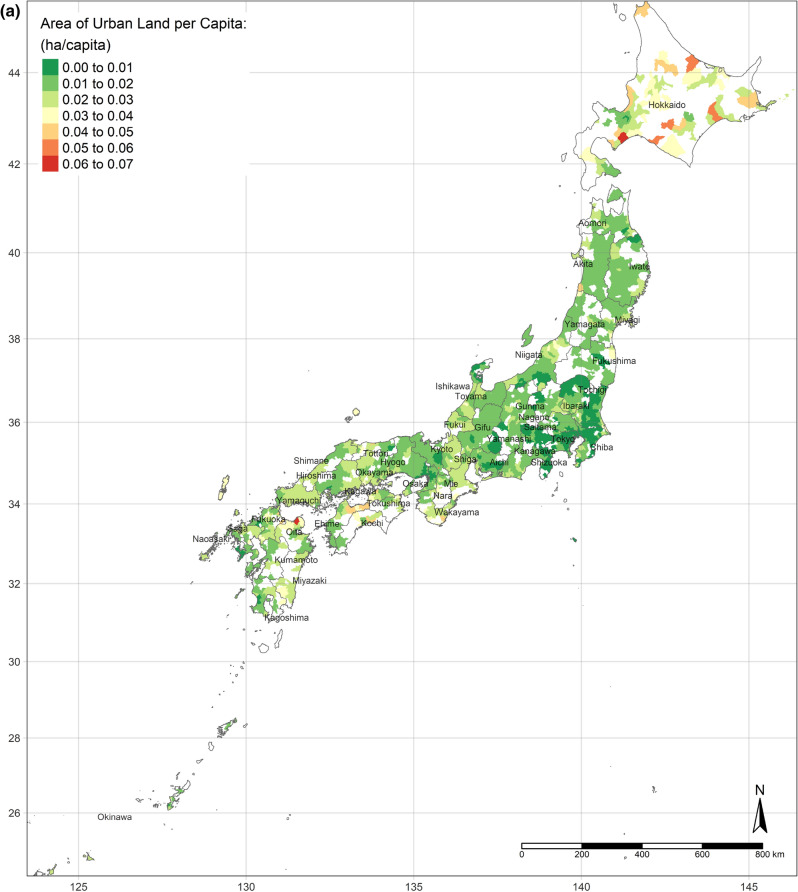

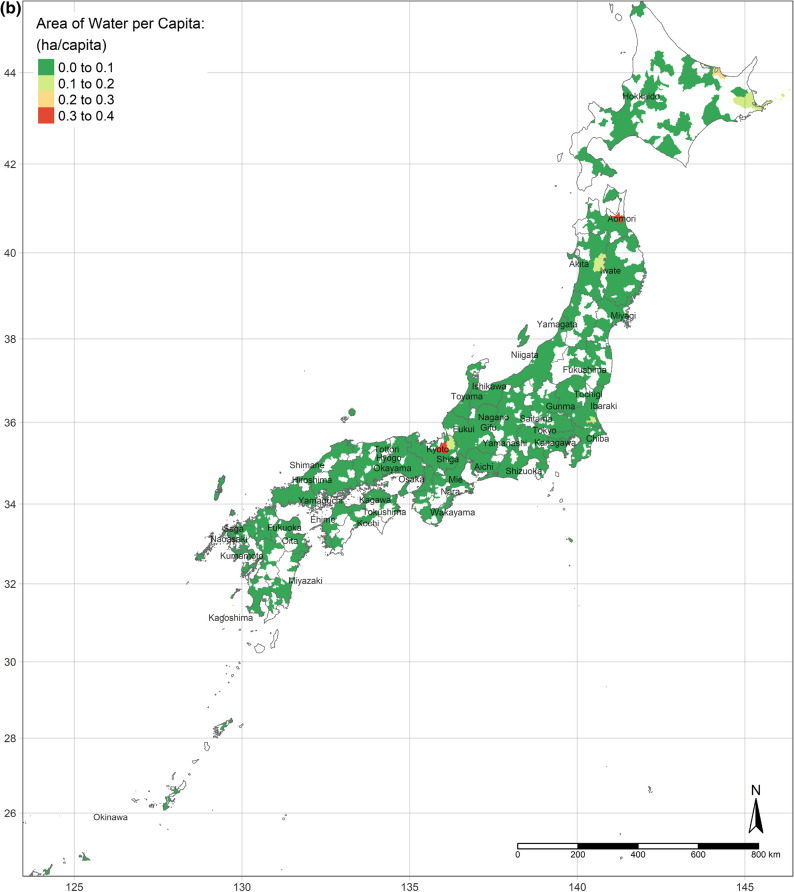

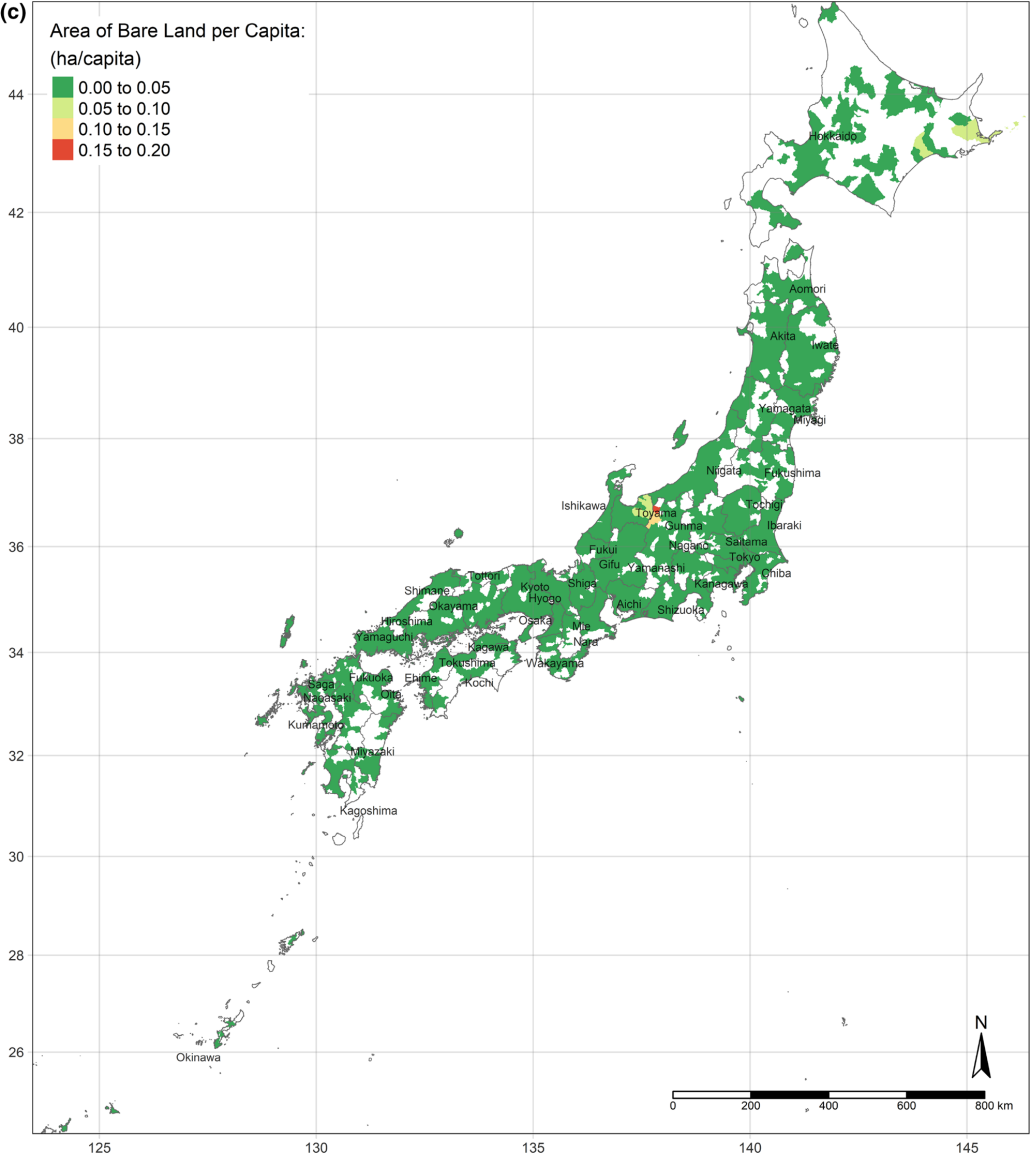
(**a**) Area of urban land per capita. (**b**) Area of water per capita. (**c**) Area of bare land per capita. (Figure a, b, c is created by R 4.0.4, https://cran.r-project.org/bin/windows/base/old/4.0.4/).

The GWR model obtains spatial distributions of estimated parameters of all independent variables. According to the OLS results and the standard errors of each variable from the GWR model, the results of the areas of water, urban land, and bare land per capita are concentrated. There is no apparent nationwide difference between the areas of water and bare land per capita, whereas the area of urban land per capita is definitely less in highly developed areas (shown as Fig. 2a–c).

**Figure 3:**
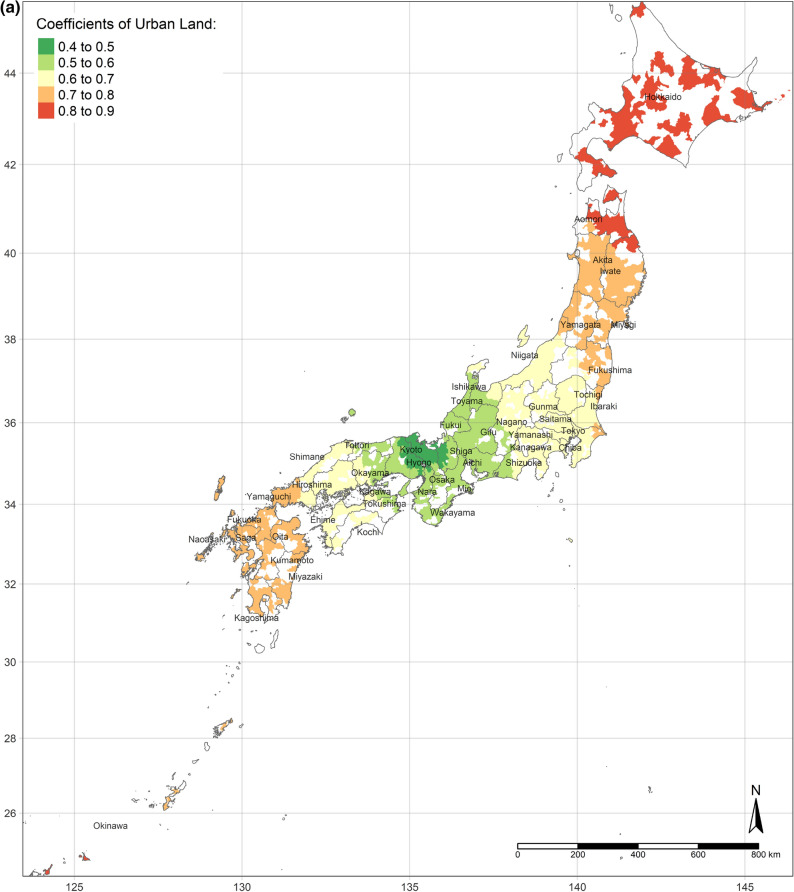

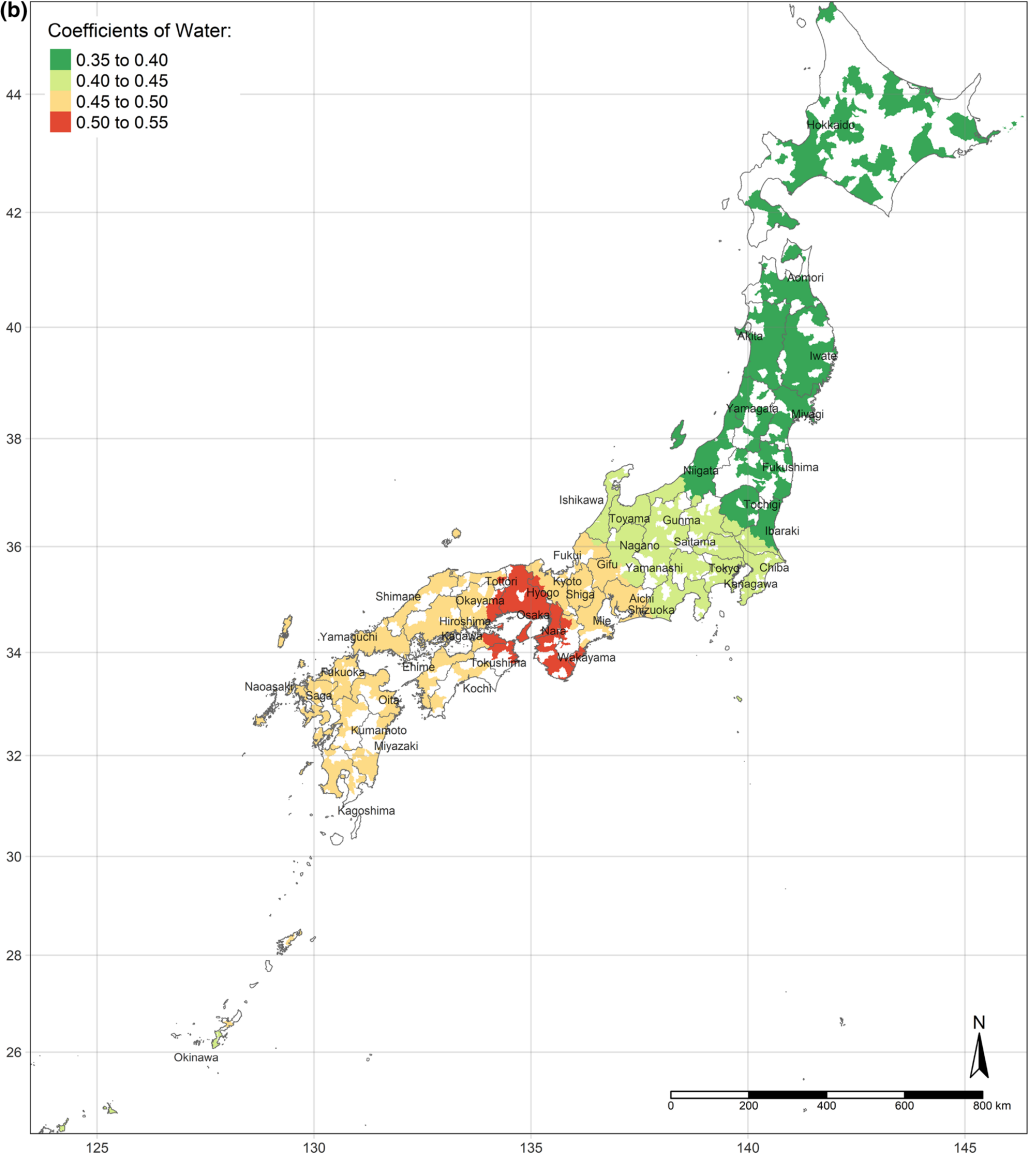

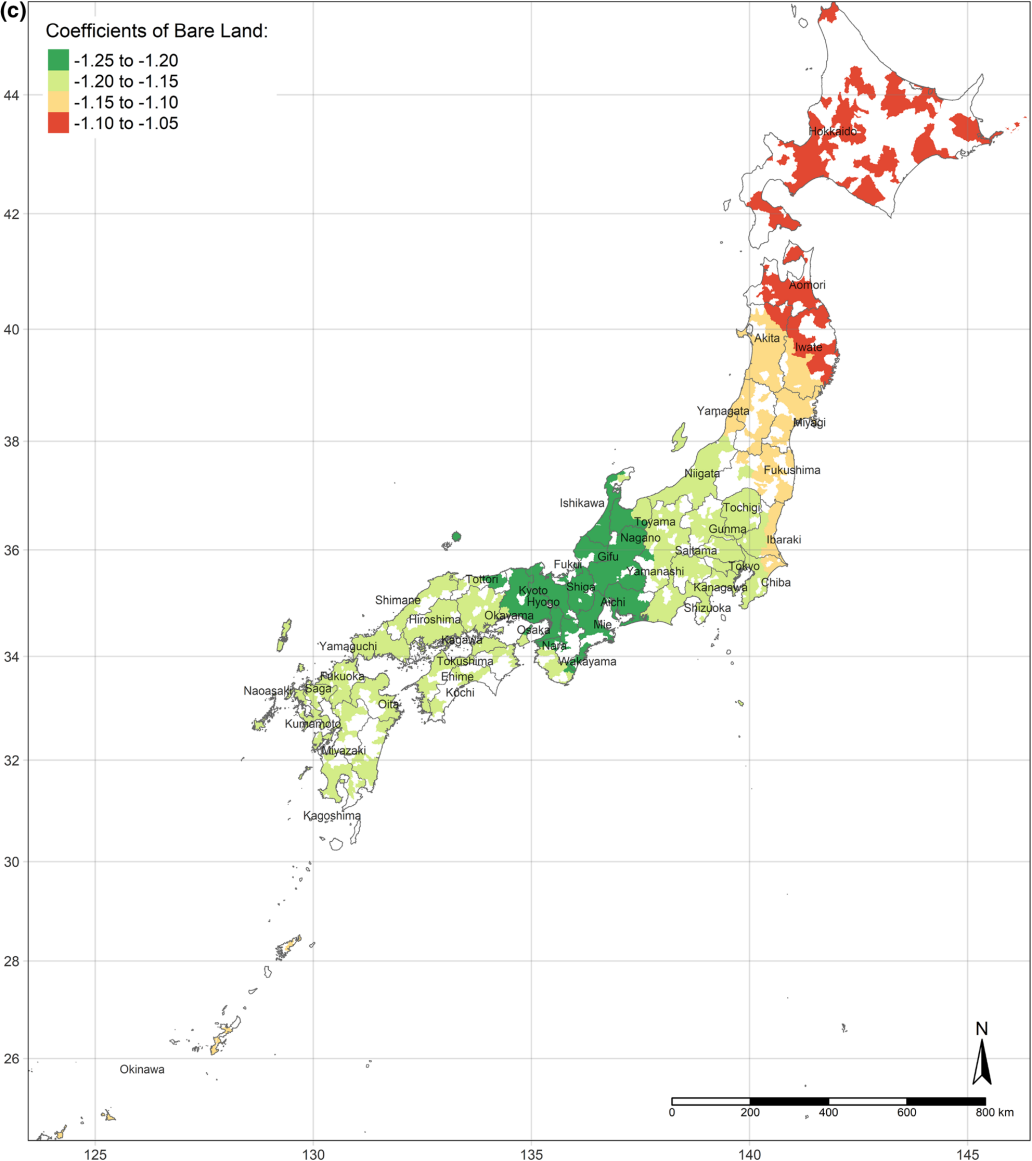
(**a**) Coefficients of urban land. (**b**) Coefficients of water. (**c**) Coefficients of bare land. (Figure a, b, c is created by R 4.0.4, https://cran.r-project.org/bin/windows/base/old/4.0.4/).

The effect of an increase in urban land on SWB is the lowest in central Japan, and demonstrates gradient increment toward Southern and Northern (Fig. 3a). Interestingly, the effect of urban land is the positively highest, where its area per capita is the most. Moreover, the coefficients of urban land are always positive, though they vary depending on locations. This situation is consistent with the results of the OLS and SLX models overall. Likewise, according to the spatial distributions of the coefficients of the area of water and bare land per capita, water and bare land also have the lowest effects on SWB in central Japan, while they have the strongest effects in the northernmost (the Hokkaido Island) and southernmost (the Kyushu Island), either positively or negatively (Fig. 3b,c). (Spatial distributions of the standard errors of urban land, water, and bare land from the GWR model are shown by Figure S1-S3; local R^2^ of each sub-prefecture region are illustrated in Figure S4.) These three spatial distributions of coefficients implicitly indicate that people living in Hokkaido Island and Kyushu Island are more sensitive to land cover change in a way.

Table 2 illustrates the MWTPs of several land types, estimated by the SLX model, though we also use the OLS model’s estimation as the comparison. Specifically, a 1 ${\mathrm{m}}^{2}$ increase in the area of urban land per capita in a sub-prefecture region is equivalent to a roughly 35,760-Japanese Yen (equivalent to 345.68 United States Dollars) increase in individual annual income of all the people in the region. Likewise, the MWTPs of areas of water, crops, and bare land per capita, which are 102.1 $\mathrm{USD}/\mathrm{Capita }{\mathrm{m}}^{2}$, − 29.82 $\mathrm{USD}/\mathrm{Capita }{\mathrm{m}}^{2}$ and − 267.51 $\mathrm{USD}/\mathrm{Capita }{\mathrm{m}}^{2}$, can be explained in the same way. In addition, the result from the SLX model also shows that a city’s SASWB is also associated with the area of grassland per capita in its neighbor cities. In other words, a 1 ${\mathrm{m}}^{2}$ increase in the area of grassland per capita in its neighbor cities is equivalent to a roughly 12.77 USD increase in the individual annual income of all the people living in it.

**Table 2:** MWTP of land cover.

Land type	Coefficients	MWTP	95%CI	p value
**SLX model**				
Area of water per capita	0.37	102.1	23.4 to 180.79	0.011
Area of urban land per capita	1.27	345.68	51.1 to 640.26	0.021
Area of crops per capita	− 0.11	− 29.82	− 63.51 to 3.87	0.083
Area of bare land per capita	− 0.98	− 267.51	− 515.95 to 19.08	0.035
Area of grassland per capita in neighbor cities	0.05	12.77	1.37 to 24.17	0.028
**OLS model**				
Area of water per capita	0.41	96.44	31.32 to 161.57	0.004
Area of urban land per capita	0.85	199.34	18.34 to 380.34	0.031
Area of bare land per capita	− 1.09	− 255.13	− 427.89 to 82.37	0.004

The land cover variables without significant coefficients are not shown.

Exchange rate in 2016 from World Bank is used, where 1 United States Dollars (USD) is roughly equal to 103.45 Japanese Yen: https://data.worldbank.org/indicator/PA.NUS.PPP.

Unit of MWTP: $\frac{\mathrm{USD}}{\mathrm{Capita}} {m}^{2}.$

**Figure 4:**
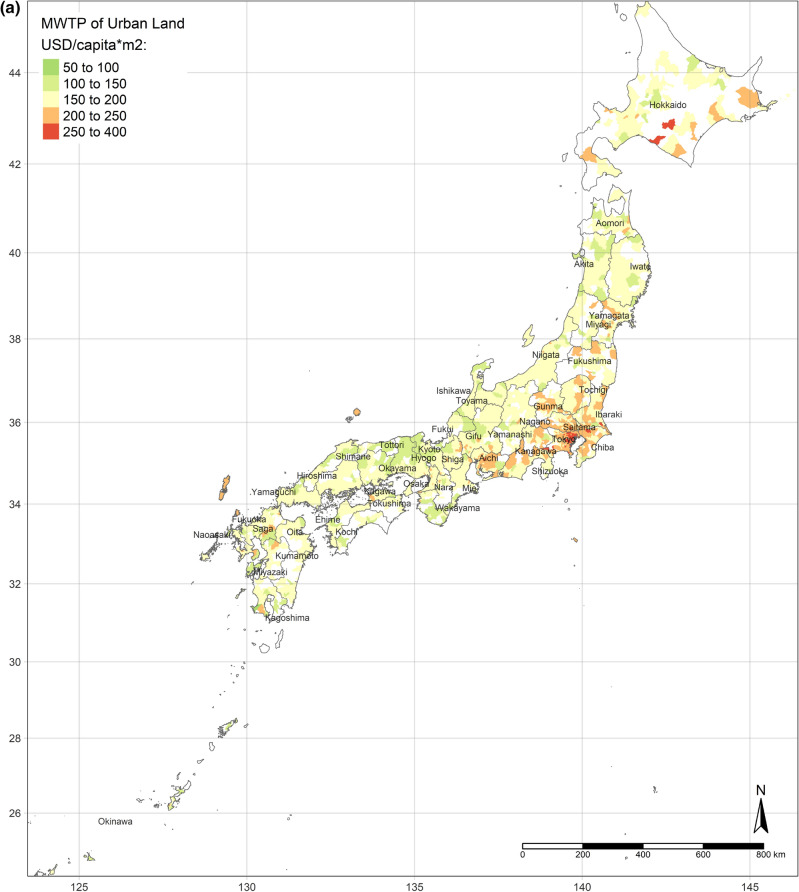

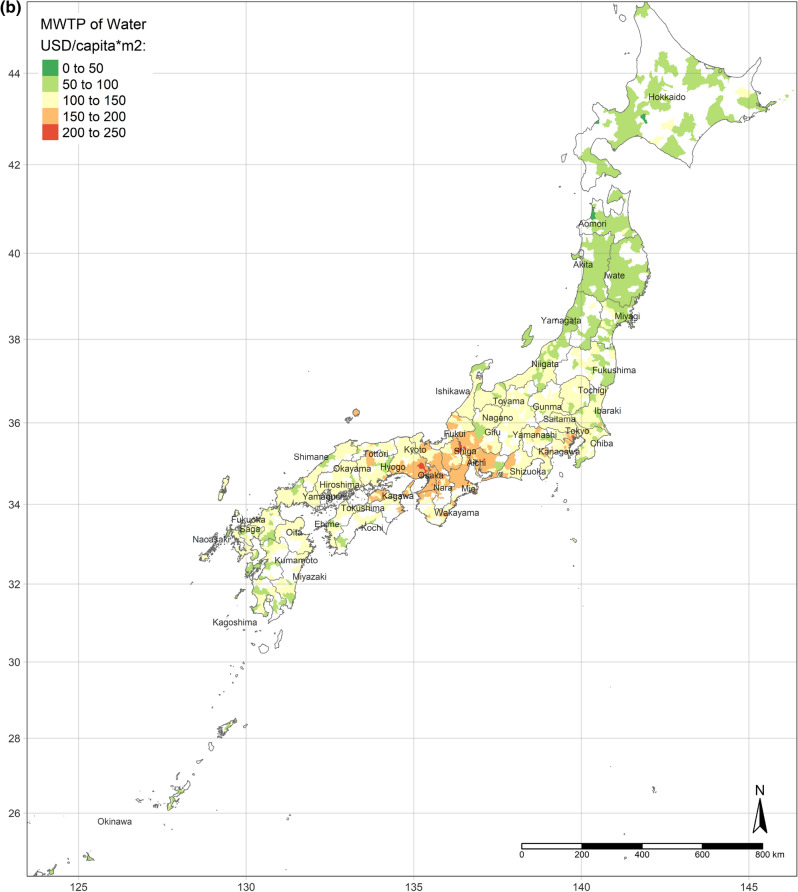

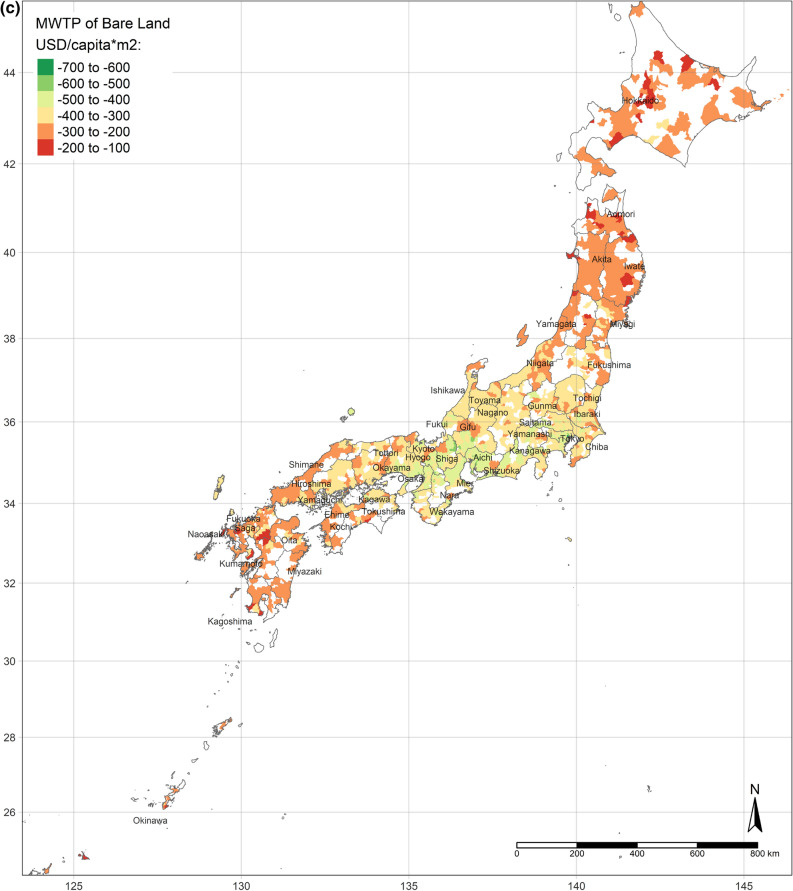
(**a**) MWTP of urban land. (**b**) MWTP of water. (**c**) MWTP of bare land. (Figure a, b, c is created by R 4.0.4, https://cran.r-project.org/bin/windows/base/old/4.0.4/).

Figure 4a shows the spatial distribution of the MWTP of urban land, based on the GWR model. Compared with Fig. 2a, the MWTP is higher in the area with the lower area of urban land per capita, consistent with scarcity value theory. In fact, Tokyo is a point in case. Tokyo has the most total area of urban land, but the area per capita is lower than the mean. Additionally, the MWTP of urban land in Tokyo is the highest. Figure 4b,c are illustrated the spatial distribution of the MWTPs of water and bare land. People living in central Japan are willing to pay more for water land cover, while the MWTP of water land cover of people in Hokkaido is the lowest. Similar to the spatial distribution of water, people in central Japan also have the most negative attitude toward bare land, while the MWTP in Hokkaido is higher, though still negative.

According to the analyses, urban land, water, and bare land are significantly associated with human well-being in all models. It also demonstrates that the monetary value of urban land on human well-being is the highest, while the monetary value of bare land is the most negative. Interestingly, grassland in a region is not significantly associated with its human well-being but related to its neighbors’. Moreover, our results illustrate the spatial variability of the relationships between land cover and human well-being. According to the GWR model, the monetary value of urban land in highly developed areas is the highest, according to the spatial model.

As the robustness checks, first, we compare all coefficients from four global models, including OLS, SAR, SEM, SLX (illustrated in Table S3). The coefficients of the four global models are similar. Moreover, the local coefficients of each subsample are in the direction of the global models. Secondly, we perform cross-validation in OLS. The R^2^ of the test sample (20% of the total sample) is 44.5%, similar to the R^2^ (49.1%) using the total sample. The result of cross-validation indicates the reliability of the models with selected variables. Here, we cannot perform cross-validation in the spatial models because random division in the total sample would cause changes in the spatial contexts of the sub-prefecture regions. To further check the robustness of the SLX model, we use another SWB indicator, happiness, to substitute for life satisfaction. In this robustness check, there is no dramatic change in the significance or value of the parameters. Because the GWR has already divided the total sample into several subsamples, we can check its robustness by analyzing the statistical distributions of each coefficient of the subsamples. According to this robustness check, the coefficients of critical variables are relatively similar. Thus, the main models in our analyses are robust.

## Discussion

This article analyzes the relationship between land cover and human well-being using spatial models. Unprecedentedly, our research concentrate on the city-level data converted from individual-level data. Our study analyzes the monetary values of urban land and other land types, taking spatial contexts into account, and provides informative ideas on urbanization for sustainable development.

Urban land is positively associated with human well-being. In our regression models, we obtain a significantly positive coefficient of urban land. Previous work indicates that the urban land, road, and mine are positively related to the SWB in other places^6^. The possible explanations for the anticipation of more urban land are as follows: first, larger home sizes, more entertainment, better environment, among others, are desired to improve human well-being, as the individual income increases^10,16,33^. According to the previous studies, the relationship between home size and SWB is positive^16^. Additionally, parks and other kinds of urban greenery are positively correlated with human well-being^5,33^. For example, the monetary values of greenery in the residential area and roadsides in the Great Tokyo Area are 12,102 JPY and 55,707 JPY (around 116.98 USD and 538.49 USD), respectively^5^. To increase both home size and urban greenery, an increase in urban land is a necessary option. Naturally living in cities is an effective way to improve human well-being when cities have sufficient urban land^13^. Secondly, land cover change, including the built-up area, affects poverty^24^. Insufficient urban land is negatively associated with the wealth index^24^. Besides, in the light of inclusive wealth theory, the increase in urban land raises the human capital and manufacture capital in Japan, exceeding the loss of natural capital^54,55^. Thirdly, urban congestion would become more serious in the future, which adversely impacts human well-being. Because the Japanese urbanization rate is still increasing^1^, more people might concentrate in the cities, especially metropolitan areas. Moreover, the relationship between individual income and human well-being is also positive. Previous studies show that income helps people meet their basic and psychological demands, which matters to human well-being to a point^10,42^. According to LSA, monetary values is the marginal rate of substitution between land cover change and income in this study. Because both urban land and income positively affect human well-being, the marginal rate of substitution is positive. In our study, for urban land, the monetary value is estimated at 345.68 $\mathrm{USD}/\mathrm{Capita }{\mathrm{m}}^{2}$.

Water and grassland in neighbor cities are positively associated with human well-being. Previous studies point out that momentary SWB is higher when people are in natural land types, including water^16,27,56^. Water and grasslands make humans feel happy because of attention restoration, artistic inspiration and improving health^13,30^. Similar to urban land, the monetary values of water and grassland in neighbor cities are estimated at 102.1 $\mathrm{USD}/\mathrm{Capita }{\mathrm{m}}^{2}$ and 12.77 $\mathrm{USD}/\mathrm{Capita }{\mathrm{m}}^{2}$, respectively. However, bare land and crops are negatively related to human well-being, consistent with previous works^6,27,33^. Previous studies indicate that bare land, like abandoned land, adversely impacts emotion^27^, causes unsafe feelings and fear of crime^34^. Crops are negatively related to infectious disease risks, which adversely affect human well-being^57^. Because of people’s preferences, the monetary values of crops and bare land are calculated at − 29.82 $\mathrm{USD}/\mathrm{Capita }{\mathrm{m}}^{2}$ and − 267.51 $\mathrm{USD}/\mathrm{Capita }{\mathrm{m}}^{2}$, respectively.

Additionally, the relationships between land cover and human well-being are affected by the spatial contexts. Previous studies illustrate the spatial variability of the relationship between SWB and land cover by grouped regressions or difference-in-differences^6^ and MWTPs for specific land types tend to be higher when they are scarcer^5,6^. In our study, the MWTP of urban land is higher in highly developed areas due to the scarcity of urban land (shown in Fig. 2a). In other words, in highly developed areas, the urban land per capita is relatively lower than in small cities. Besides, the individual annual income is higher in highly developed areas, such as Great Tokyo, Osaka, and Nagoya. Owing to higher individual income and more opportunities, such metropolitan areas attract more people to come^58^. The congestion issue will become more severe in metropolitan areas in the future. These regions need to either build more urban land or evacuate the population to improve human well-being. Oppressiveness and danger are associated with crowded environments, which adversely affect human well-being^59^. According to our research, the reason for the congestion of big cities is that the cities are not big enough compared to their population sizes. The spatial distribution of the coefficient of water on human well-being is similar to the coefficient of urban land, while it is opposite to the coefficient of bare land. It could be explained that people dislike bare land more, where they prefer urban land and water more. Compared with previous individual-level research, this study provides more information for the governments, who can control and lead land cover optimization. Because the purpose of this research is to provide a basis for the government’s land-use policy, the city-level analyses are more effective than the individual-level research, rather than ecological fallacy. According to the analyses results, urban land, water, and grassland are preferred, while bare land and crops are disliked. However, these situations are always ignored by the public. Land-use policies, such as tax policies, urban planning, among others, could optimize the land cover patterns to improve human well-being by converting disliked land types to desired land types. Based on the spatial analyses, the land-use policies should be spatially different.

Several limitations are worthy of note. First, our analyses are using cross-sectional data, so the difference within observations is ignored. Because panel land cover dataset cannot be obtained, we are unable to detect temporal variability of the relationship between human well-being and land cover. Secondly, the sampling of the survey is spatially uneven. There are more people sampled in highly developed areas compared with the situation in rural regions. Finally, although four spatial models are applied in our research, the spatial heterogeneity might still strongly affect the analyses because the reality is far more complicated than the models we assumed. In future studies, models’ performance could be improved by employing more variables, such as mental health, number of family members, emotions, meteorological data, air pollution data, among others. Moreover, model optimization is needed. In this study, the linear relationships are assumed even in spatial models, but the relationships might be more complicated. Other methods combining spatial models and machine learning technology, such as artificial neural network or random forest, may greatly improve the goodness of fit.

## Conclusions

Urban land is positively associated with human well-being. Specifically, a 1 ${\mathrm{m}}^{2}$ increase in the area of urban land per capita in a city is equivalent to an approximately 345.68 USD increase in the individual annual income of all the people in the city. The monetary values of areas of water, crops, and bare land per capita, which are 102.1 $\mathrm{USD}/\mathrm{ Capita }{\mathrm{m}}^{2}$, − 29.82 $\mathrm{USD}/\mathrm{Capita }{\mathrm{m}}^{2}$ and − 267.51 $\mathrm{USD}/\mathrm{Capita }{\mathrm{m}}^{2}$, respectively. Intriguingly, grassland in a region is not associated with its human well-being, but related to its neighbors’. Moreover, in the highly developed areas, the monetary value of urban land is actually the highest, based on the GWR model. To conclude, our research provides valuable insights into urbanization on human well-being for the policymaker to formulate effective land-use policies.

## Supplementary Information

Supplementary Information 1.
