# Supplementary material for: Land cover matters to human well-being
Source: Sci Rep. 2021 Aug 5;11:15957. doi: 10.1038/s41598-021-95351-6 (PMC8342716; doi:10.1038/s41598-021-95351-6)
Supplement: Supplementary file 1 — Supplementary Information 1. [file 41598_2021_95351_MOESM1_ESM.docx]

Supplementary Materials for

Land Cover Matters to Human Well-Being

Chao Li^1^, Shunsuke Managi*^1^

^1^ Urban Institute & School of Engineering, Kyushu University, Japan

* Correspondent to: Shunsuke Managi, [managi@doc.kyushu-u.ac.jp](mailto:managi@doc.kyushu-u.ac.jp), Kyushu University 744 Motooka, Nishi-ku, Fukuoka 819-0395 Japan

# Supplementary Materials

| **Table S1: Data Statistic Summary** | | | | | |
| --- | --- | --- | --- | --- | --- |
|  | **N** | **Mean** | **St. Dev.** | **Min** | **Max** |
| **SASWB** | 1,234 | 3.352 | 0.134 | 2.780 | 3.844 |
| **Area of water per capita (ha/capita)** | 1,234 | 0.006 | 0.022 | 0.000 | 0.366 |
| **Area of Urban Land per capita (ha/capita)** | 1,234 | 0.017 | 0.009 | 0.002 | 0.065 |
| **Area of Crops per capita (ha/capita)** | 1,234 | 0.031 | 0.066 | 0.000 | 1.166 |
| **Area of Rice Paddy per capita (ha/capita)** | 1,234 | 0.035 | 0.045 | 0.000 | 0.393 |
| **Area of Grassland per capita (ha/capita)** | 1,234 | 0.035 | 0.188 | 0.000 | 5.604 |
| **Area of DBF per capita (ha/capita)** | 1,234 | 0.117 | 0.328 | 0.000 | 3.738 |
| **Area of DNF per capita (ha/capita)** | 1,234 | 0.051 | 0.185 | 0.000 | 2.923 |
| **Area of EBF per capita (ha/capita)** | 1,234 | 0.042 | 0.094 | 0 | 1 |
| **Area of ENF per capita (ha/capita)** | 1,234 | 0.112 | 0.232 | 0.000 | 2.226 |
| **Area of Bare Land per capita (ha/capita)** | 1,234 | 0.005 | 0.009 | 0.00003 | 0.174 |
| **Individual Annual Income (1 Million JPY)** | 1,234 | 4.186 | 0.703 | 2.150 | 7.719 |
| **Frequency of High Levels Stress** | 1,234 | 3.171 | 0.158 | 2.156 | 3.843 |
| **Frequency of Low Levels Stress** | 1,234 | 3.576 | 0.136 | 2.992 | 4.294 |
| **Ease of Stress Relief** | 1,234 | 3.252 | 0.141 | 2.567 | 3.938 |
| **Living Environment Comfort** | 1,234 | 3.654 | 0.249 | 2.406 | 4.353 |
| **Living Environment Safety** | 1,234 | 2.963 | 0.169 | 2.167 | 3.588 |
| **Community Attachment** | 1,234 | 3.273 | 0.175 | 2.611 | 4.000 |
| **Self-Reported Health** | 1,234 | 3.228 | 0.140 | 2.567 | 3.864 |
| **Percentage of Student (%)** | 1,234 | 1.123 | 1.215 | 0.000 | 10.526 |
| **Percentage of Company Owner (%)** | 1,234 | 2.232 | 2.043 | 0.000 | 18.421 |
| **Percentage of Government Officer (%)** | 1,234 | 6.130 | 4.599 | 0.000 | 40.476 |
| **Percentage of Self-employed (%)** | 1,234 | 0.078 | 0.045 | 0.000 | 0.312 |
| **Percentage of Professional Job (%)** | 1,234 | 2.530 | 2.083 | 0.000 | 17.647 |
| **Percentage of Housewife (%)** | 1,234 | 10.268 | 4.444 | 0.000 | 34.286 |
| **Percentage of Retired (%)** | 1,234 | 6.458 | 3.960 | 0.000 | 27.869 |
| **Percentage of Unemployed (%)** | 1,234 | 7.211 | 3.649 | 0.000 | 25.000 |
| **Percentage of College without Diploma (%)** | 1,234 | 22.369 | 6.784 | 0.000 | 56.923 |
| **Percentage of Bachelor (%)** | 1,234 | 37.632 | 9.967 | 0.000 | 81.081 |
| **Percentage of Master (%)** | 1,234 | 3.415 | 2.820 | 0.000 | 16.981 |
| **Percentage of Ph.D. (%)** | 1,234 | 1.017 | 1.503 | 0.000 | 15.000 |
| **Percentage of Male (%)** | 1,234 | 48.472 | 1.400 | 42.757 | 57.774 |
| **Percentage Of Population 45-64 (%)** | 1,234 | 58.476 | 4.047 | 46.283 | 74.843 |
| **Percentage Of Population >= 65 (%)** | 1,234 | 28.290 | 5.406 | 14.896 | 45.674 |
| **Population Density (10,000/km2)** | 1,234 | 0.230 | 0.375 | 0.001 | 2.238 |

| **Table S2: Data Sources and Detailed Information of Variables** | | |
| --- | --- | --- |
|  | Data Sources | Detialed Information |
| **SASWB** | Survey | To acauire individual SWB, the question is "Overall, how satisfied are you with your life?". Answers is from 5, Completely satisfied, to 1, Completely dissatisfied. |
| **Area of water per capita (ha/capita)** | Nationwide land cover dataset with 30 meters resolution in 2015 from Japan Aerospace Exploration Agency <https://www.eorc.jaxa.jp/ALOS/en/lulc/lulc_index.htm> |  |
| **Area of Urban Land per capita (ha/capita)** |  |  |
| **Area of Crops per capita (ha/capita)** |  |  |
| **Area of Rice Paddy per capita (ha/capita)** |  |  |
| **Area of Grassland per capita (ha/capita)** |  |  |
| **Area of DBF per capita (ha/capita)** |  |  |
| **Area of DNF per capita (ha/capita)** |  |  |
| **Area of EBF per capita (ha/capita)** |  |  |
| **Area of ENF per capita (ha/capita)** |  |  |
| **Area of Bare Land per capita (ha/capita)** |  |  |
| **Individual Annual Income (1 Million JPY)** | Survey | The respondents should select one range of income. Moreover, the midpoint of the selected range are considered as the individual annual income. |
| **Frequency of High Levels Stress** | Survey | The question is "how often do you feel high levels of stress in your daily life?" . Answer is from 5, always, to 1, never. |
| **Frequency of Low Levels Stress** | Survey | The question is "how often do you feel low levels of stress in your daily life?" . Answer is from 5, always, to 1, never. |
| **Ease of Stress Relief** | Survey | The question is "do you think stress relief and mood change are well done?" . Answer is from 5, very essay, to 1, very difficult. |
| **Living Environment Comfort** | Survey | The question is "do you feel comfort about the living environment you live in?" . Answer is from 5, very comfortable, to 1, very uncomfortable. |
| **Living Environment Safety** | Survey | The question is "do you feel safe in the community you live in?" . Answer is from 5, very safe, to 1, very dangerous. |
| **Community Attachment** | Survey | The question is "do you attach the community you live in?" . Answer is from 5, very attached, to 1, not attached at all. |
| **Self-Reported Health** | Survey | The question is "how is your health overall?" . Answer is from 5, very healthy, to 1, very unhealthy. |
| **Percentage of Student (%)** | Survey |  |
| **Percentage of Company Owner (%)** | Survey |  |
| **Percentage of Government Officer (%)** | Survey |  |
| **Percentage of Self-employed (%)** | Survey |  |
| **Percentage of Professional Job (%)** | Survey |  |
| **Percentage of Housewife (%)** | Survey |  |
| **Percentage of Retired (%)** | Survey |  |
| **Percentage of Unemployed (%)** | Survey |  |
| **Percentage of College without Diploma (%)** | Survey |  |
| **Percentage of Bachelor (%)** | Survey |  |
| **Percentage of Master (%)** | Survey |  |
| **Percentage of Ph.D. (%)** | Survey |  |
| **Percentage of Male (%)** | Statistic data from japanese governent <https://www.e-stat.go.jp/stat-search?page=1> |  |
| **Percentage Of Population 45-64 (%)** | Statistic data from japanese governent <https://www.e-stat.go.jp/stat-search?page=1> |  |
| **Percentage Of Population >= 65 (%)** | Statistic data from japanese governent <https://www.e-stat.go.jp/stat-search?page=1> |  |
| **Population Density (10,000/km2)** | Statistic data from japanese governent <https://www.e-stat.go.jp/stat-search?page=1> Sub-prefecture regions' boundaries from Jananese Ministry of Land, Infrastructure, Transport and Tourism <https://nlftp.mlit.go.jp/ksj/index.html> |  |

| **Table S3: Full Regressions Results** | | | | | | |
| --- | --- | --- | --- | --- | --- | --- |
|  | Dependent variable: SASWB | | | | | |
|  | **OLS Model** | **SAR Model** | **SEM Model** | | **SLX Model** | |
|  |  |  |  | X | | Lag X |
| **Area of water per capita (ha/capita)** | 0.411^***^ | 0.411^***^ | 0.425^***^ | 0.374^**^ | | -0.069 |
|  | (0.141) | (0.139) | (0.141) | (0.147) | | (0.187) |
| **Area of Urban Land per capita (ha/capita)** | 0.849^**^ | 0.846^**^ | 0.879^**^ | 1.266^**^ | | -0.443 |
|  | (0.393) | (0.387) | (0.403) | (0.550) | | (0.742) |
| **Area of Crops per capita (ha/capita)** | -0.078 | -0.078 | -0.085^*^ | -0.109^*^ | | 0.119 |
|  | (0.050) | (0.050) | (0.051) | (0.063) | | (0.087) |
| **Area of Rice Paddy per capita (ha/capita)** | 0.060 | 0.062 | 0.063 | 0.155 | | -0.115 |
|  | (0.082) | (0.081) | (0.083) | (0.102) | | (0.151) |
| **Area of Grassland per capita (ha/capita)** | 0.013 | 0.013 | 0.004 | -0.001 | | 0.047^**^ |
|  | (0.020) | (0.019) | (0.020) | (0.021) | | (0.021) |
| **Area of DBF per capita (ha/capita)** | 0.005 | 0.005 | 0.006 | 0.005 | | -0.005 |
|  | (0.013) | (0.013) | (0.013) | (0.015) | | (0.022) |
| **Area of DNF per capita (ha/capita)** | 0.016 | 0.016 | 0.015 | 0.003 | | 0.037 |
|  | (0.022) | (0.022) | (0.023) | (0.026) | | (0.040) |
| **Area of EBF per capita (ha/capita)** | 0.045 | 0.043 | 0.044 | 0.050 | | -0.007 |
|  | (0.039) | (0.039) | (0.040) | (0.055) | | (0.076) |
| **Area of ENF per capita (ha/capita)** | -0.004 | -0.004 | -0.005 | -0.008 | | -0.002 |
|  | (0.017) | (0.017) | (0.018) | (0.021) | | (0.030) |
| **Area of Bare Land per capita (ha/capita)** | -1.086^***^ | -1.086^***^ | -1.020^***^ | -0.980^**^ | | -0.992 |
|  | (0.375) | (0.370) | (0.381) | (0.464) | | (0.680) |
| **Individual Annual Income (1 Million JPY)** | 0.017^***^ | 0.017^***^ | 0.017^***^ | 0.015^**^ | | 0.003 |
|  | (0.006) | (0.006) | (0.006) | (0.006) | | (0.011) |
| Frequency of High Levels Stress | -0.039 | -0.039 | -0.039 | -0.030 | | -0.030 |
|  | (0.027) | (0.027) | (0.027) | (0.027) | | (0.051) |
| Frequency of Low Levels Stress | -0.063^**^ | -0.063^**^ | -0.061^**^ | -0.066^**^ | | -0.025 |
|  | (0.027) | (0.027) | (0.027) | (0.027) | | (0.053) |
| Ease of Stress Relief | 0.349^***^ | 0.348^***^ | 0.341^***^ | 0.346^***^ | | 0.143^***^ |
|  | (0.027) | (0.027) | (0.027) | (0.028) | | (0.051) |
| Living Environment Comfort | 0.145^***^ | 0.145^***^ | 0.147^***^ | 0.140^***^ | | -0.042 |
|  | (0.021) | (0.021) | (0.021) | (0.022) | | (0.037) |
| Living Environment Safety | -0.003 | -0.003 | 0.007 | 0.029 | | -0.173^***^ |
|  | (0.024) | (0.023) | (0.024) | (0.026) | | (0.042) |
| Community Attachment | 0.094^***^ | 0.094^***^ | 0.087^***^ | 0.094^***^ | | 0.129^***^ |
|  | (0.022) | (0.022) | (0.022) | (0.023) | | (0.043) |
| Self-Reported Health | 0.088^***^ | 0.089^***^ | 0.086^***^ | 0.083^***^ | | 0.015 |
|  | (0.023) | (0.023) | (0.023) | (0.023) | | (0.048) |
| Percentage of Student (%) | 0.003 | 0.003 | 0.003 | 0.004^*^ | | 0.004 |
|  | (0.002) | (0.002) | (0.002) | (0.002) | | (0.005) |
| Percentage of Company Owner (%) | -0.002 | -0.002 | -0.002 | -0.002 | | -0.005^*^ |
|  | (0.001) | (0.001) | (0.001) | (0.001) | | (0.003) |
| Percentage of Government Officer (%) | 0.00001 | -0.00005 | 0.0001 | -0.0001 | | -0.001 |
|  | (0.001) | (0.001) | (0.001) | (0.001) | | (0.001) |
| Percentage of Self-employed (%) | -0.083 | -0.086 | -0.080 | -0.083 | | -0.015 |
|  | (0.072) | (0.071) | (0.071) | (0.073) | | (0.143) |
| Percentage of Professional Job (%) | 0.0003 | 0.0002 | 0.0004 | 0.001 | | -0.005 |
|  | (0.001) | (0.001) | (0.001) | (0.001) | | (0.003) |
| Percentage of Housewife (%) | 0.002^***^ | 0.002^***^ | 0.002^***^ | 0.002^***^ | | -0.001 |
|  | (0.001) | (0.001) | (0.001) | (0.001) | | (0.001) |
| Percentage of Retired (%) | -0.001^*^ | -0.001^*^ | -0.001 | -0.002^**^ | | -0.003^**^ |
|  | (0.001) | (0.001) | (0.001) | (0.001) | | (0.002) |
| Percentage of Unemployed (%) | -0.003^***^ | -0.003^***^ | -0.003^***^ | -0.003^***^ | | -0.003^*^ |
|  | (0.001) | (0.001) | (0.001) | (0.001) | | (0.002) |
| Percentage of College without Diploma (%) | 0.001^**^ | 0.001^**^ | 0.001^**^ | 0.001^*^ | | 0.0001 |
|  | (0.0005) | (0.0005) | (0.0005) | (0.0005) | | (0.001) |
| Percentage of Bachelor (%) | 0.001^*^ | 0.001^*^ | 0.001^*^ | 0.001 | | 0.001 |
|  | (0.0004) | (0.0004) | (0.0004) | (0.0004) | | (0.001) |
| Percentage of Master (%) | 0.003^**^ | 0.003^**^ | 0.003^**^ | 0.003^**^ | | -0.002 |
|  | (0.001) | (0.001) | (0.001) | (0.001) | | (0.002) |
| Percentage of Ph.D. (%) | -0.002 | -0.002 | -0.002 | -0.002 | | -0.003 |
|  | (0.002) | (0.002) | (0.002) | (0.002) | | (0.004) |
| Percentage of Male (%) | 0.005^**^ | 0.005^**^ | 0.005^**^ | 0.004 | | -0.001 |
|  | (0.003) | (0.003) | (0.003) | (0.003) | | (0.004) |
| Percentage Of Population 45-64 (%) | -0.006^**^ | -0.006^**^ | -0.006^**^ | -0.006^**^ | | 0.0004 |
|  | (0.002) | (0.002) | (0.002) | (0.003) | | (0.003) |
| Percentage Of Population >= 65 (%) | -0.001 | -0.001 | -0.001 | -0.001 | | 0.0004 |
|  | (0.002) | (0.002) | (0.002) | (0.002) | | (0.002) |
| Population Density (10,000/km2) | -0.043^***^ | -0.043^***^ | -0.041^***^ | -0.044^**^ | | -0.024 |
|  | (0.012) | (0.011) | (0.012) | (0.021) | | (0.027) |
| Constant | 1.442^***^ | 1.454^***^ | 1.432^***^ | 1.450^***^ | | |
|  | (0.307) | (0.304) | (0.306) | (0.331) | | |
| Observations | 1,234 | 1,234 | 1,234 | 1,234 | | |
| R^2^ | 0.491 |  |  | 0.520 | | |
| Adjusted R^2^ | 0.476 |  |  | 0.492 | | |
| Log Likelihood |  | 1,148.993 | 1,151.103 |  | |  |
| sigma^2^ |  | 0.009 | 0.009 |  | |  |
| Akaike Inf. Crit. | -2225.843 | -2,223.985 | -2,228.206 | -2229.969 | | |
| Residual Std. Error | 0.097 (df = 1199) |  |  | 0.095 (df = 1165) | | |
| F Statistic | 33.987^***^ (df = 34; 1199) |  |  | 18.536^***^ (df = 68; 1165) | | |
| Wald Test (df = 1) |  | 0.143 | 5.281^**^ |  | |  |
| LR Test (df = 1) |  | 0.142 | 4.363^**^ |  | |  |
| Note: | ^*^p < 0.1, ^**^p < 0.05, ^***^p < 0.01 | | | | | |
| X in the SLX model represents the variables of sub-prefecture regions themselves, while Lag X represents the variables of their neighbors. | | | | | | |





Figure S1: Standard Error of the Local Coefficients of Urban Land

(Figure S1 is created by R 4.0.4, https://cran.r-project.org/bin/windows/base/old/4.0.4/)





Figure S2: Standard Error of the Local Coefficients of Water

(Figure S2 is created by R 4.0.4, https://cran.r-project.org/bin/windows/base/old/4.0.4/)





Figure S3: Standard Error of the Local Coefficients of Bare Land

(Figure S3 is created by R 4.0.4, https://cran.r-project.org/bin/windows/base/old/4.0.4/)





Figure S4: Spatial Distribution of Local R^2^

(Figure S4 is created by R 4.0.4, https://cran.r-project.org/bin/windows/base/old/4.0.4/)
